# Sensitivity of Human Papillomavirus (HPV) Lineage and Sublineage Variant Pseudoviruses to Neutralization by Nonavalent Vaccine Antibodies

**DOI:** 10.1093/infdis/jiz401

**Published:** 2019-08-14

**Authors:** Anna Godi, Troy J Kemp, Ligia A Pinto, Simon Beddows

**Affiliations:** 1 Virus Reference Department, Public Health England, London, United Kingdom; 2 HPV Serology Laboratory, Frederick National Laboratory for Cancer Research, Maryland, USA

**Keywords:** human papillomavirus, vaccine, variant, lineage, antibody, neutralization

## Abstract

Natural variants of human papillomavirus (HPV) are classified into lineages and sublineages based upon whole-genome sequence, but the impact of diversity on protein function is unclear. We investigated the susceptibility of 3–8 representative pseudovirus variants of HPV16, HPV18, HPV31, HPV33, HPV45, HPV52, and HPV58 to neutralization by nonavalent vaccine (Gardasil®9) sera. Many variants demonstrated significant differences in neutralization sensitivity from their consensus A/A1 variant but these were of a low magnitude. HPV52 D and HPV58 C variants exhibited >4-fold reduced sensitivities compared to their consensus A/A1 variant and should be considered distinct serotypes with respect to nonavalent vaccine-induced immunity.

Human papillomavirus (HPV) is the causative agent of cervical and other cancers, accounting for >600 000 cases globally per annum [1]. Replication of the small double-stranded DNA genome is facilitated by host polymerases resulting in a low evolutionary rate, although distinct genotypes have arisen over time [2]. Genotypes from the *Alphapapillomavirus* genus are associated with the development of cervical cancer [1], with HPV16 conferring the highest relative risk.

Whole-genome sequence analysis has led to the delineation of distinct variant lineages and sublineages that exhibit both geographical bias in their distribution and differential disease risk [2–4]. Efforts are also underway to understand the evolution of HPV variants from their prehistoric origins [3, 4]. HPV16 [3] and HPV58 [4] non-A lineages (B/C/D) are estimated to have split from their respective lineage A viruses approximately 400–600 thousand years ago, followed by the further resolution of lineages B, C, and D approximately 100–200 thousand years ago, coincident with the evolution and migration of ancient hominins from Neanderthals/Denisovans to modern humans [3, 4].

The HPV capsid is an icosahedral lattice comprising 72 pentamers of the major capsid protein (L1) and includes the asymmetrical and/or stochastic distribution of the minor capsid protein (L2). Neutralizing antibodies target the L1 capsid and can passively protect in preclinical infection models leading to the development of highly efficacious L1 virus-like particle (VLP)-based prophylactic vaccines [5]. Bivalent (Cervarix®) and quadrivalent (Gardasil®) vaccines target the most prevalent oncogenic genotypes (HPV16 and HPV18) while the nonavalent (Gardasil®9) HPV vaccine targets 5 additional oncogenic genotypes (HPV31, HPV33, HPV45, HPV52, and HPV58). Quadrivalent and nonavalent vaccines also target HPV6 and HPV11, which can cause genital warts.

The biological consequences of HPV genome variants are unclear. Studies examining the potential impact of natural variation on L1 antigenicity have examined variants of HPV16 [6, 7], HPV31 [8], HPV33 [9], HPV45 [10], HPV52 [11], and HPV58 [12] and demonstrated, in some cases, variant-specific differences in sensitivity to monovalent, bivalent, and quadrivalent VLP immune sera, natural infection sera, and monoclonal antibodies (MAbs). Here, we evaluate the sensitivity of lineage and sublineage variants to neutralization by nonavalent HPV vaccine antibodies.

## METHODS

### Ethics Statement

Serum samples, representing a source of nonavalent vaccine antibodies, were obtained with consent from the donor and where applicable their legal guardian.

### Vaccine Serum Samples

Eighteen donors who received at least 1 dose of Gardasil®9 were evaluated in this study (Supplementary Table 1). The majority of individuals were white (56%), male (61%), and received at least 2 doses of vaccine (83%). Samples were collected a median 175 (interquartile range [IQR], 79–377; n = 17) days after the last dose of vaccine. The median age of the donors at sample collection was 14 years (IQR, 12–17 years). Donors were collected from 3 sources: BioIVT (formally BioreclamationIVT, Hicksville, NY), Bio|Options (Brea, CA), and Occupational Health Services (Frederick National Laboratory for Cancer Research, Frederick, MD). Serum was stored at −80°C.

### L1L2 Variant Pseudoviruses

Codon-optimized L1 and L2 genes representing consensus lineage and sublineage variant sequences (Supplementary Figure 1) were synthesized (GeneArt; Thermo Fisher Scientific) with additional site-directed mutagenesis (QuikChange Site-Directed Mutagenesis Kit, Agilent Technologies) as required [8–12]. Inserts were confirmed by Sanger sequencing. Bicistronic psheLL vectors containing these inserts were assembled and with a luciferase reporter (pGL4.51 [luc2/CMV/Neo]; Promega) used to transfect 293TT cells, as previously described [12].

The pseudovirus (PsV) neutralization assay was performed as previously described [12]. A standardized input of 300 TCID_50_ was used for all PsVs. The neutralizing antibody titer was assigned as the reciprocal of the serum dilution that resulted in an 80% reduction in the luciferase signal compared to control wells (PsV and cells only), estimated by interpolation. For analysis purposes, serum titers less than the lower limit of detection (LOD, 50) were assigned a censored value of 25. For each genotype, a single serial dilution series of each serum was tested against all relevant variant PsVs in parallel. The titer of each serum against each variant PsV was compared to that generated against the consensus A/A1 variant PsV and a fold difference metric was generated to allow normalization and intra-/intergenotype comparisons [8–12].

To demonstrate reproducibility, samples were retested against 8 variant PsV (HPV16 A; HPV31 A1, A2, B1; HPV52 A1, D; HPV58 A1, C) resulting in a Pearson correlation coefficient of 0.970 and a median ratio of 1.00 (IQR, 0.98–1.02; n = 75) for the resulting paired log_10_ titers. The median ratio of the fold differences for the variants of HPV31 (A2, B1), HPV52 (D), and HPV58 (C) compared to their respective A1 consensus variant was 1.0 (IQR, 0.7–1.2; n = 36) for the initial and repeat data.

### Statistical Analysis

Wilcoxon signed-rank test was used to compare neutralization titers between each HPV variant and the designated consensus A/A1 PsV (Stata 15; StataCorp, College Station, TX).

## RESULTS

### Representative Variant PsVs

**Figure 1.**
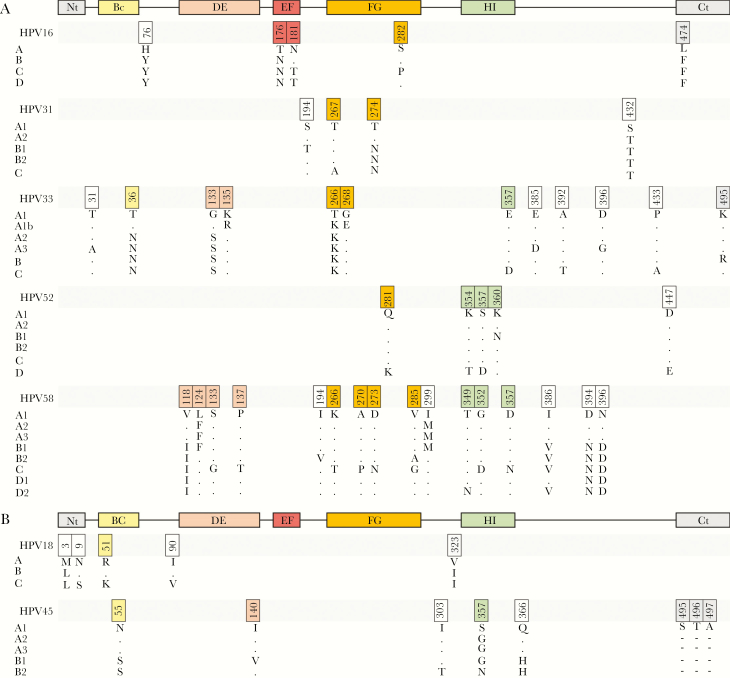
Consensus lineage and sublineage variant pseudovirus major capsid protein map. Consensus L1 sequences used for the creation of lineage and sublineage representative pseudoviruses are shown (Supplementary Figure 1). Variants residues within the external surface exposed loops (BC, DE, EF, FG, and HI) are highlighted as are the N and C termini (Nt, Ct). Dots depict identity with the A/A1 consensus pseudovirus and indels are highlighted as dashes. Sequences are aligned within species group for (*A*) alpha-9 genotypes (HPV16, HPV31, HPV33, HPV52, and HPV58) and (*B*) alpha-7 genotypes (HPV18 and HPV45). Numbering is genotype-specific according to the indicated genome reference for each genotype (Supplementary Figure 1) starting with the second methionine according to convention (https://pave.niaid.nih.gov/).

DNA sequence fragments (approximately 2.9 kb) encoding the L1 and L2 capsid proteins for each genotype were assembled (July–October 2018) (Supplementary Figure 1). Consensus lineage and sublineage protein sequences were derived where possible using the majority residue at each variant position. Representative variant PsV have previously been created for HPV31, HPV33, HPV45, HPV52, and HPV58 [8–12]. For the present study, lineage variants of HPV16 and HPV18, additional sublineage variants of HPV31 (HPV31 A1, A2, B1, B2) [8], and updated A1 and D2 sublineage variants of HPV58 [12] were created. For HPV16 and HPV18 the consensus lineage A was used as the benchmark sequence while the consensus sublineage A1 was used for the remaining genotypes (Figure 1). The distribution of sublineages at the level of translated L1 and L2 protein sequences did not always reflect the number of sublineage variants defined by their whole-genome sequence and more weight was put upon variant residues within L1 as this protein is the target for neutralizing antibodies elicited against L1 antigens. In many cases, the complete genome reference sequence (typically used for nucleotide numbering) and the commonly used L1L2 pseudovirus sequences did not fully represent contemporary A/A1 consensus sequences (Supplementary Figure 1).

### Sensitivity of Variant PsVs to Nonavalent Vaccine Antibodies

**Figure 2.**
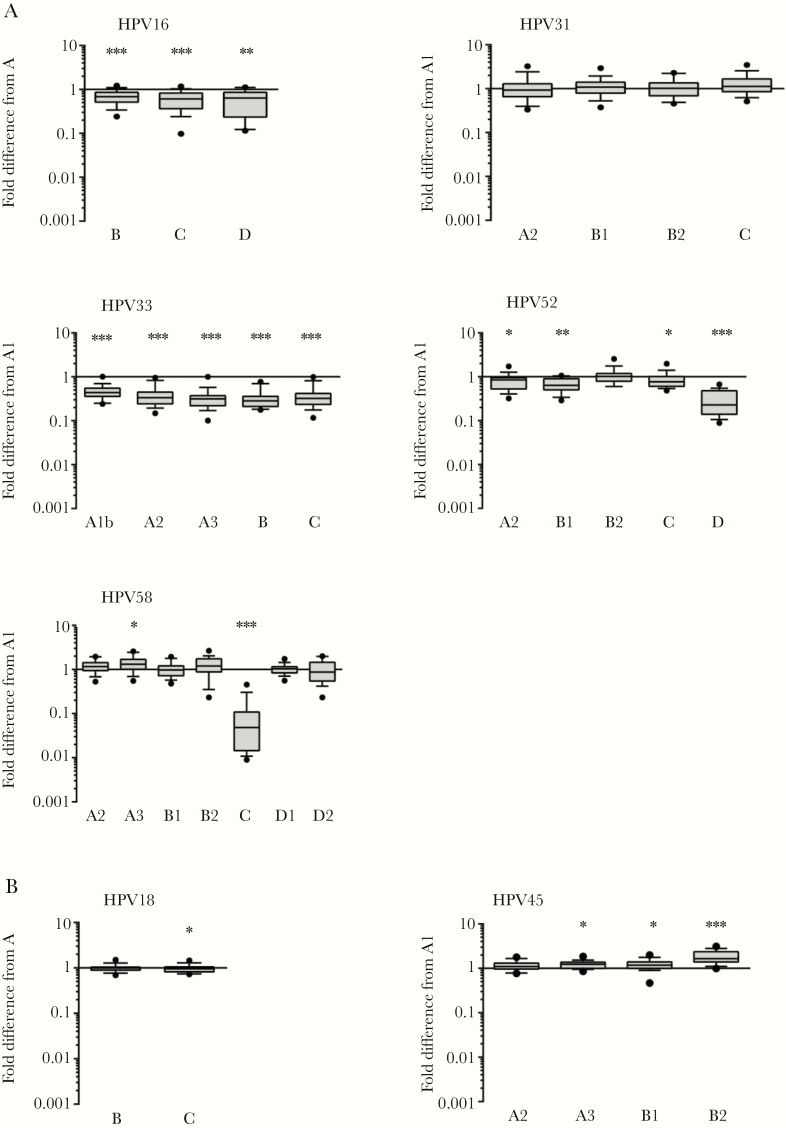
Neutralization of variant pseudoviruses by nonavalent vaccine antibodies. Fold difference in neutralization titers between each representative consensus lineage or sublineage pseudovirus and the consensus A (HPV16 and HPV18) or A1 (HPV31, HPV33, HPV45, HPV52, and HPV58) pseudovirus are shown. Boxes represent median and IQR, and whiskers 10th and 90th percentiles. *, *P* < .05; **, *P* < .01; ***; *P* < .001 (Wilcoxon signed-rank test).

Nonavalent vaccine sera (n = 18) were tested for neutralizing antibodies against variant PsVs (Figure 2). One or more PsV variants of HPV16, HPV18, HPV33, HPV45, HPV52, and HPV58 displayed altered susceptibility to nonavalent vaccine antibodies, but these were mostly within 2-fold of the A/A1 consensus PsV. Variant PsVs of HPV31 displayed similar sensitivity to the HPV31 A1 consensus PsV. All HPV33 variant PsVs displayed 2- to 4-fold lower sensitivity to nonavalent vaccine antibodies compared to the HPV33 consensus A1 PsV (Supplementary Table 2). HPV52 lineage D PsV (median titer 1859; IQR, 660–16 091) exhibited a median 4.4-fold (IQR, 2.2–6.8) reduction in sensitivity compared to HPV52 sublineage A1 PsV (median titer 11 945; IQR, 4425–48 402). HPV58 lineage C PsV (median titer 100; IQR, 25–757) displayed a median 21-fold (IQR, 10–64) reduced sensitivity to nonavalent vaccine antibodies compared to the HPV58 consensus A1 PsV (median titer 5090; IQR, 1760–12 795). The sera in this study displayed a wide range of titers against each A/A1 reference PsV, exemplified by the wide IQR, although this degree of imprecision was reduced when normalized to a pairwise fold difference metric.

## DISCUSSION

Our understanding of the extent of HPV genome variation has improved significantly due to the recent application of whole-genome sequencing, including definitive variant nomenclature (designation of lineages and sublineages), improved resolution of variant disease association, global dispersal, and prehistorical origins [2–4]. The aim of the present study was to evaluate the potential for naturally occurring polymorphisms within the L1 and L2 genes of lineage and sublineage variants of nonavalent vaccine-incorporated genotypes to impact capsid antigenicity and susceptibility to neutralization by nonavalent vaccine antibodies.

Natural polymorphisms in HPV16 L1 residues affect susceptibility to some L1-specific MAbs [7], but these make little difference to neutralization sensitivity to polyclonal human or animal immune sera raised against HPV16 L1 VLP [6, 7]. In the present study, HPV16 lineage variants B, C, and D exhibited slightly (<2-fold) reduced sensitivity to nonavalent vaccine sera compared to lineage A, suggesting that variant residues in the EF and FG loops and the Ct have little antigenic impact on recognition by nonavalent vaccine antibodies. There are no published data on whether HPV18 variants exhibit differential sensitivity to L1-specific neutralizing antibodies, but in the present study HPV18 lineage variants A, B, and C displayed similar sensitivity to nonavalent vaccine sera. HPV31 FG loop polymorphisms can affect the neutralization sensitivity of some L1-specific MAbs, but neutralization sensitivity to antibodies derived by bivalent or quadrivalent vaccination or natural infection were similar [8]. Similarly, type-specific nonavalent vaccine antibodies elicited against HPV31 were not affected by these natural capsid polymorphisms. HPV33 variants displayed a reduced (2- to 4-fold) sensitivity to type-specific nonavalent vaccine sera similar to that seen with cross-reactive bivalent and quadrivalent vaccine sera [9], suggesting that variant residues in the DE and FG loops alter capsid antigenicity to a range of antibody specificities. Natural variation in the HPV45 L1 capsid bestows an increased (A2, A3, B1) or reduced (B2) sensitivity to cross-reactive bivalent and quadrivalent vaccine antibodies [10]. In the present study, nonavalent vaccine antibodies demonstrated little (<2-fold) or no difference in the recognition of variants A2, A3, B1, and B2. These data suggest that antibodies elicited following HPV18 VLP immunization that cross-recognize HPV45 comprise different specificities to the type-specific antibodies elicited by HPV45 VLP in the nonavalent HPV vaccine. Most HPV52 (A2, B1, B2, and C) and HPV58 (A2, A3, B1, B2, D1, and D2) variants exhibited little (<2-fold) or no difference in neutralization sensitivity compared to their respective sublineage A1, whilst HPV52 lineage D and HPV58 lineage C exhibited a >4-fold reduced sensitivity to neutralization by nonavalent vaccine antibodies. The differential recognition of HPV52 D and HPV58 C by nonavalent vaccine sera is in keeping with previously published data on differential sensitivity to natural infection antibodies, preclinical antisera, and L1-specific MAbs for these genotypes [11, 12]. Furthermore, these data suggest that HPV52 D and HPV58 C variants are antigenically distinct from the other lineages within their respective genotypes and should be considered distinct serotypes with respect to nonavalent vaccine-induced immunogenicity.

It is not clear whether reduced susceptibility demonstrated in vitro will have any real-world consequence. It is feasible that in Africa, where these lineages tend to predominate [11, 12], that reduced vaccine effectiveness against HPV52 and HPV58-associated infections would be observed, perhaps in low-dose settings and years after vaccination when protective immunity wanes. For the remaining genotypes, these data suggest that nonavalent vaccine antibodies recognize global variants of the incorporate genotypes similarly. There are no efficacy data from Africa-based HPV vaccine trials or effectiveness data from postlicensure HPV vaccine programs in Africa, although these data will become available in time. Any potential impact of the differential antigenicity of HPV52 lineage D and HPV58 lineage C variants should be seen in the context of overall HPV52/58-associated disease burden (approximately 5% of total) and the prevalence of these lineages (approximately 20%–30%) within HPV52 or HPV58 infections.

Post hoc analysis of bivalent vaccine efficacy data demonstrated low-dose efficacy against incident HPV16/18 infections and low-grade disease [13], but low-dose effectiveness in the context of postlicensure immunization programs is less clear [14]. Antibodies alone can provide protection from infection and subsequent disease in animal models, but whether they represent the primary mechanism of protection in humans is uncertain [15]. A murine challenge model demonstrated that antibody levels required for protection in vivo are orders of magnitude lower than the antibody levels capable of neutralizing the same PsV in vitro, suggesting that protective antibodies may be circulating in excess in vivo. Similarly, although HPV PsVs have been used widely to monitor antibody responses to vaccines and natural infection, as well as elucidate steps in the entry process, differences between how PsVs behave in vitro and how authentic HPVs behave in vivo are uncertain, which is a limitation of all PsV-based systems. Taken together, these empirical data provide support for HPV52 lineage D and HPV58 lineage C capsid proteins being antigenically distinct within their respective genotypes, but possible implications for global health remain to be seen.

## Supplementary Data

Supplementary materials are available at *The Journal of Infectious Diseases* online. Consisting of data provided by the authors to benefit the reader, the posted materials are not copyedited and are the sole responsibility of the authors, so questions or comments should be addressed to the corresponding author.

jiz401_suppl_Supplementary_Figure_1

jiz401_suppl_Supplementary_Table_1

jiz401_suppl_Supplementary_Table_2

## Notes

***Acknowledgments.*** We are indebted to John T. Schiller and Christopher Buck (National Cancer Institute, Bethesda, MD) for access to the psheLL backbone used for the pseudovirus clones.

***Financial support.*** This work was supported by Public Health England; the National Cancer Institute, National Institutes of Health (contract number HHSN261200800001E); and by the Bill and Melinda Gates Foundation to develop and distribute qualified standardized reagents for human papillomavirus research as a service to be made available to the extramural research community.

***Potential conflicts of interest.*** All authors: No reported conflicts of interest. All authors have submitted the ICMJE Form for Disclosure of Potential Conflicts of Interest. Conflicts that the editors consider relevant to the content of the manuscript have been disclosed.
