# Supplementary material for: Sensitivity of Human Papillomavirus (HPV) Lineage and Sublineage Variant Pseudoviruses to Neutralization by Nonavalent Vaccine Antibodies
Source: J Infect Dis. 2019 Aug 14;220(12):1940–5. doi: 10.1093/infdis/jiz401 (PMC6834066; doi:10.1093/infdis/jiz401)
Supplement: jiz401_suppl_Supplementary_Figure_1 [file jiz401_suppl_supplementary_figure_1.pdf]

Supplementary Figure 1

HPV16

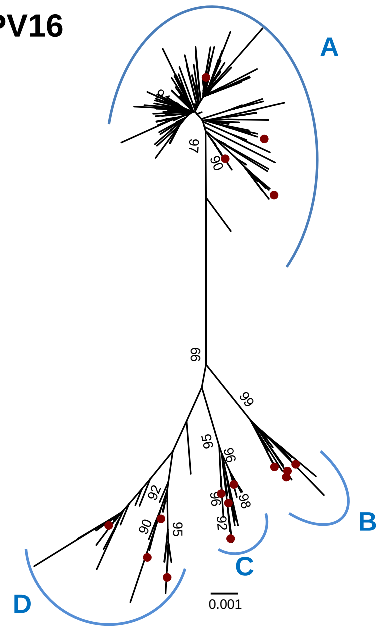

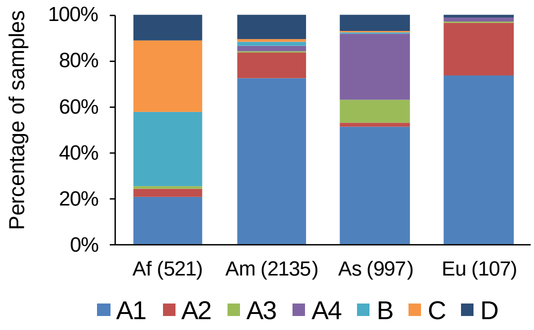

	L2												L1					
	122	245	266	269	330	352	378	384	385	420	424	443	76	176	181	282	474	*n*
**A**	**S**	**T**	**L**	**S**	**F**	**T**	**S**	**S**	**V**	**I**	**A**	**A**	**H**	**T**	**N**	**S**	**L**	*388*
B	P	.	.	P	L	A	.	.	I	T	T	G	Y	N	.	.	F	*20*
C	P	.	.	P	L	P	F	.	I	T	T	G	Y	N	T	P	F	*31*
D	P	A	F	P	L	.	V	A	I	T	T	G	Y	N	T	.	F	*62*

**Accession numbers**	NC001526; AB818687-93; AB889488-94; AF125673; AF402678; AF472508-9; AF534061; AF536179-80; AY686579-84; EU11817; EU918764; FJ006723; FJ610146-52; HM057182; HQ644234-299; JN565302-3; JQ004092-99; JQ067943-44; KC935953, KF880690, KF954093; KP212150-59; KP874716-19; KU053823-944; KU298880-85; KU641509; KU684311-14; KU684316-17; KX947269-85; KY549156-321; KY883659; KY994539; LC193821; LC368952-97
**Lineage References**	A1 (NC001526); A2 (AF536179); A3 (HQ644236); A4 (AF534061); B1 (AF536180); B2 (HQ644298); B3 (KU053910); B4 (KU053914); C1 (AF472509); C2 (HQ644644); C3 (KU053921); C4 (KU053922), D1 (HQ644257); D2 (AY686579); D3 (AF402678); D4 (KU053933)
**L1L2 consensus**	Consensus HPV16 A and PsV sequences have an Ala at residue 266 in L1 compared to a Thr in the genome reference (NC001526). In addition, HPV16 A has a Phe at residue 330 in L2 compared to a Leu in the HPV16 PsV and NC001526 sequences. HPV16 A had similar sensitivity to nonavalent sera (median 0.97; IQR 0.80-1.04; n=10; *p*=0.114) as HPV16 PsV.
**Global reference**	Clifford et al, Papillomavirus Res. 2019; 7:67-74

HPV18

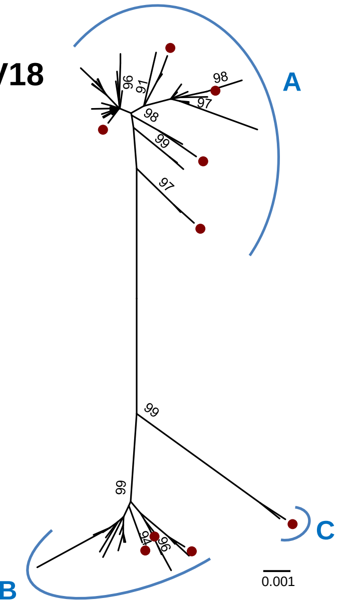

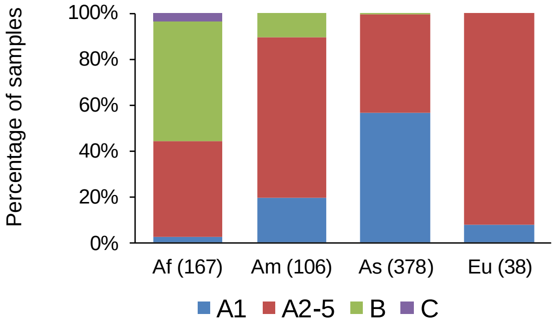

	L2															L1					
	29	33	259	266	270	328	346	355	359	365	369	370	371	373	459	3	9	51	90	323	*n*
**A**	**P**	**P**	**V**	**D**	**D**	**P**	**D**	**I**	**V**	**S**	**F**	**A**	**F**	**K**	**Y**	**M**	**N**	**R**	**I**	**V**	*71*
B	S	N	M	E	N	.	G	.	L	P	S	.	V	T	F	L	.	.	.	I	*25*
C	S	N	.	E	.	T	.	.	L	P	S	T	L	.	F	L	S	K	V	I	*2*

**Accession numbers**	NC_00135; X05015; AY262282; EF202143-55; GQ180784-85; GQ180887-08; GQ180792 KC470208-30; KU298886; KX514433; MF288652; MF288654; MF288656-58; MF288660; MF288662-65; MF288667-70; MF288672; MF288674; MF288677; MF288679-8; 0MF288682-85;, MF288687-99; MF288703-05; MF288708, MF288710; MF288712-3; MF288715; MF288717; MF288720-3; MF288726-7
**Lineage References**	A1 (AY262282); A2 (EF202146); A3 (EF202147); A4 (EF202151); A5 (GQ180787); B1 (EF202155); B2 (KC470225); B3 (EF202152); C (KC470229)
**L1L2 consensus**	Consensus HPV18 A has a Ser at residue 177, an Ile at residue 355, a Ser at residue 372 and a Tyr at residue 459 in L2 compared to an Ala, a Met and two Phe in the HPV18 PsV and in the genome reference (X05015) sequences. HPV18 A and PsV sequences have a Arg at residues 30, 283 and 338 in L1 compared to a Pro in the X05015 sequence. In addition, HPV18 A has a Met at residue 3 and an Asn at residue 88, compared to Leu and a Thr in the PsV and X05015 sequences. HPV18 A had reduced (0.41; 0.38 – 0.49; n=10; *p*=005) sensitivity to nonavalent antibodies compared to HPV18 PsV.
**Global reference**	Chen *et al.*, J. Virol. 2015; 89:10680-10687

HPV31

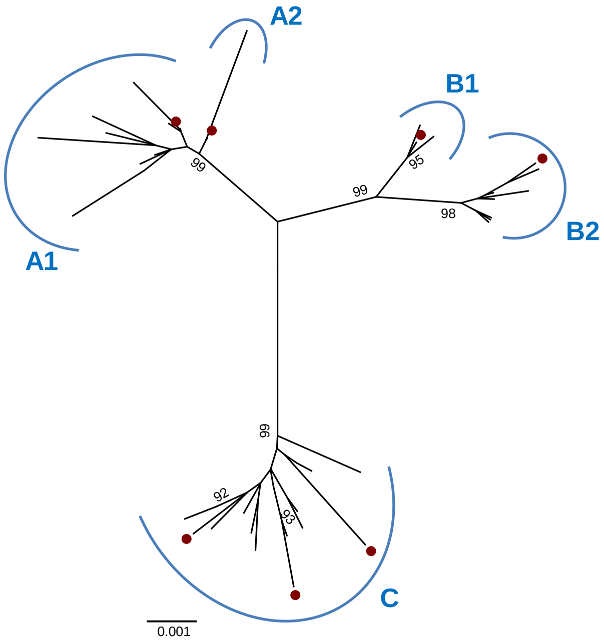

	L2				L1				
	115	260	270	377	194	267	274	432	*n*
**A1**	**V**	**S**	**I**	**V**	**S**	**T**	**T**	**S**	*15*
A2	.	T	.	.	.	.	.	T	*2*
B1	.	.	.	.	T	.	N	T	*4*
B2	.	.	.	.	.	.	N	T	*10*
C	I	.	M	L	.	A	N	T	*18*

**Accession numbers**	J04353; KX638481; KX514430; KX514424; KU298888-90; KJ754561-80; HQ537666-87
**Lineage References**	A1 (J04353); A2 (HQ537675); B1 (HQ537676); HQ537680; C1 (HQ537682); C2 (HQ537684); C3 (HQ537685)
**L1L2 consensus**	Consensus HPV31 A1 has a Ser at residue 432 in L1 compared to a Thr in the genome reference (J04353) and HPV31 PsV sequences. HPV31 A1 had similar sensitivity to nonavalent sera (1.01; 0.80-1.10; n=10; *p*=0.959) as HPV31 PsV.
**Global reference**	Limited geographical distribution data available, insufficient to plot

HPV33

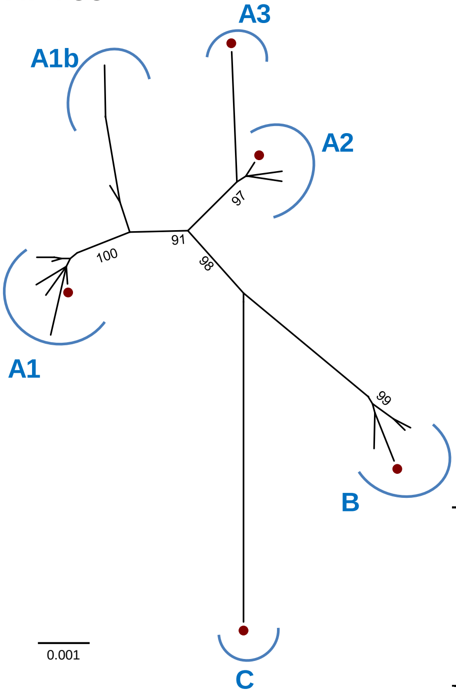

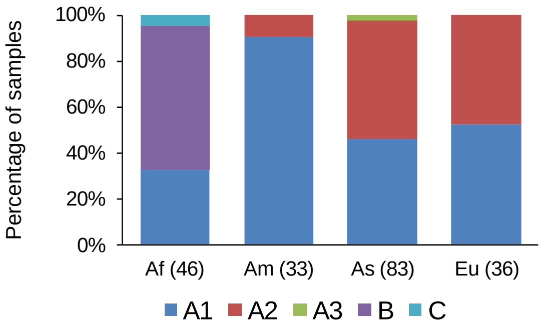

	L2											L1												
	74	77	112	131	172	195	336	350	355	360	372	31	56	133	135	266	268	357	385	392	396	433	495	*n*
**A1**	**I**	**D**	**S**	**V**	**P**	**N**	**H**	**D**		**D**	**N**	**T**	**T**	**G**	**K**	**T**	**G**	**E**	**E**	**A**	**D**	**P**	**K**	*10*
A1b	.	N	.	.	.	.	.	N	.	.	.	.	.	.	R	K	E	.	.	.	.	.	.	*3*
A2	.	N	.	.	.	.	.	H	.	N	T	.	N	S	.	K	.	.	.	.	.	.	.	*4*
A3	.	T	.	.	.	.	R	H	.	N	T	A	N	S	.	K	.	.	D	.	G	.	.	*1*
B	.	N	.	I	.	.	.	H	.	.	T	.	N	S	.	K	.	.	.	.	.	.	R	*6*
C	L	N	G	.	S	H	.	H	-	.	T	.	N	S	.	K	.	D	.	T	.	A	.	*1*

**Accession numbers**	M12732; KU298891-92; EU918766; HQ537688-707; KF436865
**Lineage References**	A1 (M12732); A2 (HQ537698); A3 (EU918766); B (HQ537705); C (KF436865)
**L1L2 consensus**	Consensus HPV33 A1, genome reference (M12732), and HPV33 PsV sequences share amino acid identity
**Global reference**	Chen et al., Virology. 2014; 448:356-362

HPV45

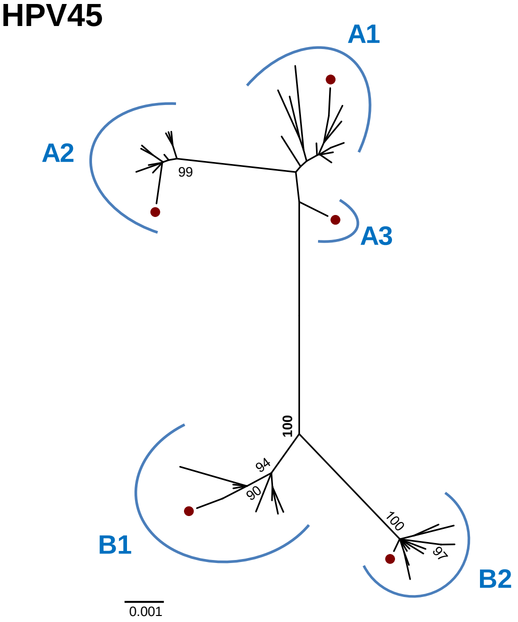

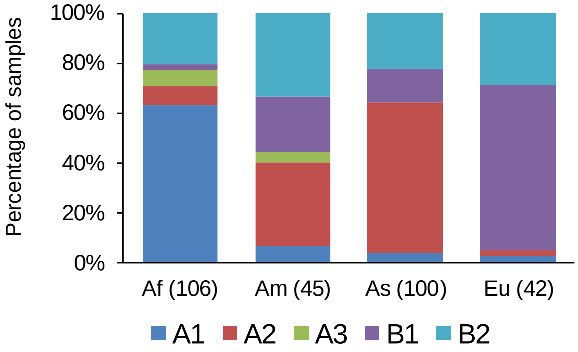

	L2									L1								
	19	43	109	114	240	244	338	424	426	55	140	303	357	366	495	496	497	*n*
**A1**	**R**	**K**	**D**	**A**	**Q**	**H**	**I**	**S**	**T**	**N**	**I**	**I**	**S**	**Q**	**S**	**T**	**A**	*15*
A2	.	.	.	.	.	.	.	.	.	.	.	.	G	.	-	-	-	*16*
A3	K	.	.	.	.	.	.	.	.	.	.	.	G	.	-	-	-	*1*
B1	K	.	E	.	R	R	L	.	S	S	V	.	G	H	-	-	-	*12*
B2	K	R	E	S	.	R	L	A	S	S	.	T	N	H	-	-	-	*16*

**Accession numbers**	X74479; DQ080002; EF202156-77; KC470250-60; KU049723-57
**Lineage References**	A1 (X74479); A2 (EF202157); A3 (KC470256); B1 (EF202161); B2 (EF202164)
**L1L2 consensus**	Consensus HPV45 A1 and PsV have an Ala at residue 222 in L2 compared to Arg in genome reference (X74479) sequence. In addition, HPV45 A1 has a Asp at residue 342 and an Asn at residue 365 compared to a Asn and a His in the HPV45 PsV and X74479 sequences. HPV45 A1 has an Asn at position 23 in L1 compared to Ser in the HPV45 PsV and X74479 sequences. HPV45 A1 had similar sensitivity to nonavalent sera (0.94; 0.87-1.01; n=10; *p*=0.333) as HPV45 PsV
**Global reference**	Chen et al., J. Virol. 2014, 88:4514-4521

HPV52

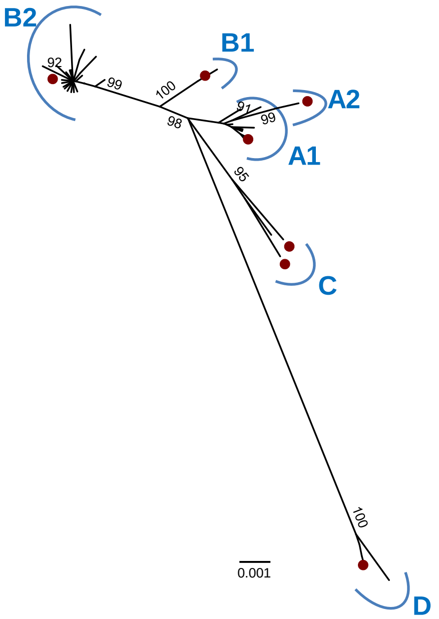

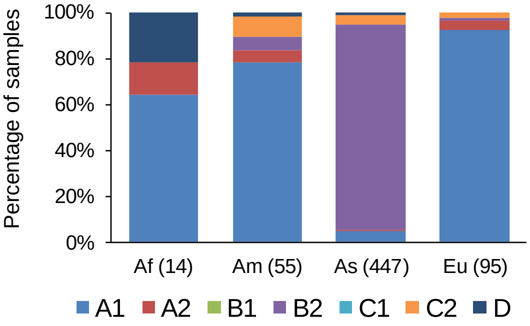

	L2							L1					
	122	169	247	272	273	274	365	281	354	357	360	447	*n*
**A1**	**S**	**I**	**Q**	**Q**	**L**	**L**	**D**	**Q**	**K**	**S**	**K**	**D**	*14*
A2	.	.	.	.	.	S	.	.	.	.	.	.	*2*
B1	.	.	.	.	.	S	.	.	.	.	N	.	*2*
B2	.	.	.	.	.	.	E	.	.	.	.	.	*59*
C	.	L	.	.	.	.	.	.	.	.	.	.	*3*
D	T	.	H	E	H	.	.	K	T	D	.	E	*5*

**Accession numbers**	X74481; AB819272-74; GQ472848; HQ537731-51; KU298906; KU298908-10; LC270024-38; LC270040-75; LC373204-7
**Lineage References**	A1 (X74481); A2 (HQ537739); B1 (HQ537740); B2 (HQ537743); C1 (HQ537744); C2 (HQ537746); D (HQ537748)
**L1L2 consensus**	Consensus HPV52 A1, genome reference (X74481), and HPV52 PsV sequences share amino acid identity
**Global reference**	Zhang et al., J Infect Dis. 2014; 210:1600-1604

HPV58

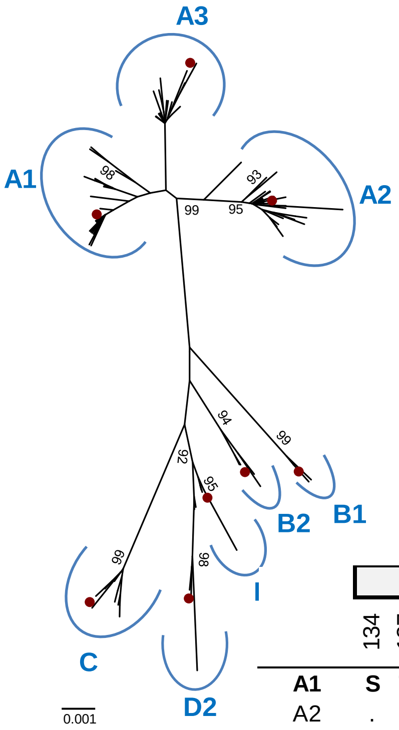

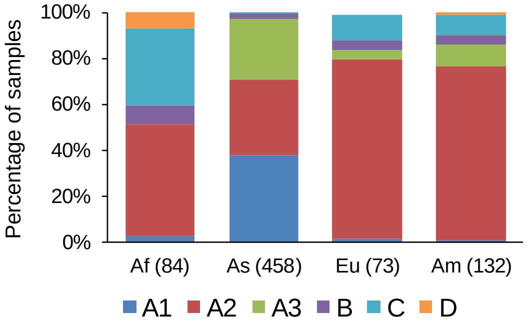

	L2														L1																
	134	137	164	169	231	342	343	352	378	381	407	417	434	447	118	124	133	137	194	266	270	273	285	299	349	352	357	386	394	396	*n*
**A1**	**S**	**T**	**T**	**L**	**N**	**Q**	**Q**	**T**	**Q**	**L**	**L**	**A**	**T**	**M**	**V**	**L**	**S**	**P**	**I**	**K**	**A**	**D**	**V**	**I**	**T**	**G**	**D**	**I**	**D**	**N**	*38*
A2	.	.	.	.	.	.	.	.	.	.	.	T	P	.	.	F	.	.	.	.	.	.	.	M	.	.	.	.	.	.	*55*
A3	.	.	.	.	T	.	.	.	.	.	.	T	.	L	.	F	.	.	.	.	.	.	.	M	.	.	.	.	.	.	*32*
B1	.	.	.	.	.	.	E	N	H	M	F	T	.	.	I	F	.	.	.	.	.	.	.	M	.	.	.	V	N	D	*3*
B2	.	.	.	.	.	.	E	.	H	M	.	T	.	.	I	.	.	.	V	.	.	.	A	.	.	.	.	V	N	D	*4*
C	.	.	.	.	.	.	E	.	H	M	.	T	.	.	I	.	G	T	.	T	P	N	G	.	.	D	N	V	N	D	*13*
D1	.	.	.	.	.	.	E	.	H	M	.	T	.	.	I	.	.	.	.	.	.	.	.	.	.	.	.	.	N	D	*5*
D2	C	I	S	F	H	E	.	H	M	.	T	.	.		I	.	.	.	.	.	.	.	.	.	N	.	.	V	N	D	*5*

**Accession numbers**	D90400; AB819275-79; EU918765; FJ385261-68; FJ407192; FJ407194-5; FJ407199-201; GQ472850; HQ537752-77; KC860269-71; KU298920; KX514422; KY225918-67; LC270076-123; LC373208-10; LC376008
**Lineage References**	A1 (D90400); A2 (HQ537752); A3 (HQ537758); B1 (HQ537762); B2 (HQ537764); C (HQ537774); D1 (HQ537768); D2 (HQ537770)
**L1L2 consensus**	Consensus HPV58 A1, genome reference (D90400), and HPV58 PsV sequences share amino acid identity
**Global reference**	Chen et al., J Virol 2017 91(21) e01285-17

Supplementary Figure 1. *Variant sequences and creation of representative pseudoviruses*
Left panel, radial Neighbor Joining tree (500 bootstrap iterations, 90% threshold) with lineage or sublineages branches indicated and appropriate representative lineage sequences highlighted (Burk et al. 2013). Right panel, geographic distribution of lineage and, where resolved, sublineage variants across four global regions (Af, Africa; Am, Americas; As, Asia; Eu, Europe) and consensus lineage and sublineage L1L2 variant sequences used for creation of representative PsV. Bottom panel, list of accession numbers used in this evaluation including differences between the genome reference, the commonly used PsV sequence and the representative consensus A/A1 used in this study. All sequences were extracted from existing sequences within the National Center for Biotechnology Information (www.ncbi.nlm.nih.gov/) database.

Burk, R. D., A. Harari and Z. Chen (2013). Human papillomavirus genome variants. Virology 445:232-243.
