# Supplementary material for: Sensitivity of Human Papillomavirus (HPV) Lineage and Sublineage Variant Pseudoviruses to Neutralization by Nonavalent Vaccine Antibodies
Source: J Infect Dis. 2019 Aug 14;220(12):1940–5. doi: 10.1093/infdis/jiz401 (PMC6834066; doi:10.1093/infdis/jiz401)
Supplement: jiz401_suppl_Supplementary_Table_1 [file jiz401_suppl_supplementary_table_1.pdf]

Supplementary Table 1. Donor characteristics

Characteristic	Measure	Outcome
Gender (n, %)	Male	11 (61%)
	Female	7 (39%)
Ethnicity (n, %)	White	10 (56%)
	Black	4 (22%)
	Mixed/Hispanic	2 (11%)
	Asian	1 (6%)
	N/A	1 (6%)
Age (Years)	Median (IQR)	14 (12 – 17)
	Range (Min – Max)	11 – 27
Vaccine doses (n, %)	1	3 (17%)
	2	7 (39%)
	3	8 (44%)

IQR, inter-quartile range; N/A, data not available
