# Supplementary material for: Sensitivity of Human Papillomavirus (HPV) Lineage and Sublineage Variant Pseudoviruses to Neutralization by Nonavalent Vaccine Antibodies
Source: J Infect Dis. 2019 Aug 14;220(12):1940–5. doi: 10.1093/infdis/jiz401 (PMC6834066; doi:10.1093/infdis/jiz401)
Supplement: jiz401_suppl_Supplementary_Table_2 [file jiz401_suppl_supplementary_table_2.pdf]

Supplementary Table 2. *Neutralizing antibody titers and fold difference from reference for lineage and sublineage PsV variants*

			Neutralizing titer*			Fold difference from A/A1 reference†				
Species	Type	Variants	Median	Q1	Q3	Median	Q1	Q3	Direction	p value
Alpha-9	16	A	9,508	4,702	51,160					
		B	5,279	3,528	37,922	0.69	0.53	0.85	↓	**<0.001**
		C	4,312	1,874	34,678	0.61	0.38	0.82	↓	**<0.001**
		D	3,048	1,806	30,833	0.64	0.24	0.86	↓	**0.002**
	31	A1	4,345	1,661	13,976					
		A2	4,393	2,174	13,201	0.93	0.70	1.27	-	0.267
		B1	5,012	2,523	16,324	1.09	0.82	1.39	-	0.913
		B2	4,326	3,140	14,441	1.02	0.71	1.29	-	0.617
		C	4,719	3,154	14,219	1.13	0.89	1.61	-	0.679
	33	A1	5,668	3,077	15,920					
		A1b	2,839	1,083	10,912	0.44	0.37	0.54	↓↓	**<0.001**
		A2	2,446	737	11,334	0.34	0.25	0.43	↓↓	**<0.001**
		A3	1,771	568	11,619	0.32	0.23	0.37	↓↓	**<0.001**
		B	2,091	805	9,710	0.28	0.22	0.35	↓↓	**<0.001**
		C	2,619	661	12,241	0.32	0.24	0.41	↓↓	**<0.001**
	52	A1	11,945	4,425	48,402					
		A2	7,270	2,525	53,139	0.85	0.55	0.91	↓	**0.039**
		B1	4,690	2,065	41,095	0.64	0.52	0.86	↓	**0.001**
		B2	10,076	3,614	57,483	1.02	0.81	1.17	-	0.983
		C	8,248	3,441	46,922	0.77	0.62	0.98	↓	**0.031**
		D	1,859	660	16,091	0.23	0.15	0.46	↓↓↓	**<0.001**
	58	A1	5,090	1,760	12,795					
		A2	7,433	1,887	13,364	1.16	0.97	1.35	-	0.170
		A3	5,673	2,186	13,614	1.31	1.03	1.66	↑	**0.035**
		B1	4,400	1,703	14,774	0.98	0.77	1.18	-	0.396
		B2	4,224	2,352	11,538	1.19	0.89	1.70	-	0.267
		C	100	25	757	0.05	0.02	0.10	↓↓↓	**<0.001**
		D1	4,820	1,981	13,479	1.03	0.86	1.12	-	0.879
		D2	4,754	1,142	9,498	0.87	0.57	1.30	-	0.112
Alpha-7	18	A	780	385	4,528					
		B	709	410	3,643	1.00	0.92	1.04	-	0.695
		C	735	419	3,343	0.96	0.84	1.02	↓	**0.040**
	45	A1	637	200	3,590					
		A2	736	202	3,380	1.10	0.98	1.30	-	0.223
		A3	812	206	3,659	1.23	1.01	1.36	↑	**0.016**
		B1	804	248	3,748	1.18	1.01	1.35	↑	**0.024**
		B2	1,449	391	5,901	1.65	1.41	2.31	↑	**<0.001**

* A total of 18 donors were evaluated for each variant.

† Arrows used to indicate direction of difference from reference: -, no significant difference; ↓ or ↑, <2 fold difference from A/A1 reference; ↓↓ or ↑↑, 2-4 fold difference from A/A1 reference; ↓↓↓ or ↑↑↑ , >4 fold difference from A/A1 reference. Note the thresholds for a 2-fold increase or decrease compared to the A/A1 reference are 2.0 and 0.5, respectively, and that of a 4-fold increase or decrease compared to the A/A1 reference are 4.0 and 0.25, respectively.
